# Pre-specified Weight Loss Before Bariatric Surgery and Postoperative Outcomes

**DOI:** 10.7759/cureus.12406

**Published:** 2020-12-31

**Authors:** Ugochukwu Chinaka, Joshua Fultang, Abdulmajid Ali, Jean Rankin, Andisheh Bakhshi

**Affiliations:** 1 General Surgery, University Hospital Ayr/University of West of Scotland, Ayr, GBR; 2 General Surgery, University Hospital Wishaw, Wishaw, GBR; 3 Midwifery and Specialist Nursing, University Of West of Scotland, Paisley, GBR; 4 School of Computing, Engineering and Physical Sciences (CEPS), University of West of Scotland, Paisley, GBR

**Keywords:** pre-specified, pre operative, weight loss and obesity, post operative, bariatric surgery, outcomes

## Abstract

Bariatric surgery is increasing exponentially to address the steep rise in the prevalence of severe obesity. Most centers require pre-specified preoperative weight loss before allowing patients to receive surgery. We examined the current evidence surrounding the potential benefits of this requirement on postoperative outcomes. We reviewed the current literature by conducting a multistage advance electronic search in Ovid®/MEDLINE® and PubMed for publications indexed after 2008 reporting preoperative weight loss and postoperative outcomes. Thirteen original publications, three randomized control trials (RCT), and five systematic reviews that met inclusion criteria were included. These were analyzed with regards to weight loss before surgery and postoperative outcomes. There were varied reports regarding the significant effect of preoperative weight loss. Six of the original articles (50%) did not identify a significant difference in the outcome while two of the RCT (essentially the same patient population, started in 2007 and reanalyzed in 2009) demonstrated some advantage. A later RCT (2012) did not show any advantage, albeit in the short term. The results of the systematic reviews, some with heterogenic designs, show no conclusive evidence that weight loss before surgery conferred improved postoperative outcomes. There is not enough high-quality evidence to back up the requirement of pre-specified preoperative weight loss before receiving surgery. Further validation of the possible benefits of pre-specified preoperative weight loss may need to be carried out.

## Introduction and background

The purpose of this article is to review available up to date evidence on pre-specified weight loss before bariatric surgery and its significance on postoperative outcomes and to identify possible gaps. Additional peri-operative outcomes regarding the duration of operation, length of hospital stay, and postoperative complications were considered. The detailed review process involved a systematic approach to searching, retrieval, and critique of the literature. 

Obesity remains a global public health concern with grave clinical consequences [1]. This chronic disease is associated with an increased risk for comorbidities, including diabetes, hypertension, sleep apnea, osteoarthritis, stroke, cardiovascular disease, and cancer. Moreover, it has been known to lead to marked reductions in self-reported quality of life [2, 3].

Surgery is the most efficacious treatment for weight loss [1]. Its sustained effectiveness exceeds other treatment modalities regarding the amount of weight loss in addition to improving associated comorbidities and quality of life [1, 4-6]. Today, most bariatric procedures are safely performed laparoscopically [2, 6].

Many bariatric services adopt a pre-specified weight loss requirement before offering surgery. Some may require people to lose about 5-10% excess weight loss before surgery, while many non-public-funded services, especially insurance companies, require active participation in a 6-12 month supervised dietary program. Others recommend a short preoperative period (not more than three weeks) of a low-calorie diet (LCD) [3, 7].

The potential advantages of pre-specified weight loss before surgery remain controversial, leading to some services withholding surgery for not meeting a certain target preoperative weight loss, even though the scientific evidence backing up such practice remains unclear [8]. The significance of the relationship between preoperative weight loss efforts and postoperative outcomes remains undetermined. Various studies have contrasting conclusions [5, 6, 8, 9]. Presently, there is no clear consensus on preoperative weight loss requirements for patients undergoing bariatric surgery [3].

## Review

Search strategy and study selection

A multistage advance electronic search of Ovid®/MEDLINE® and PubMed was conducted in December 2019 using the terms and combined terms - 'bariatric surgery' or 'gastric bypass' or 'gastroplasty', and 'weight loss' and 'preoperative and postoperative weight loss'. The search was limited to studies in the English language and those published from 2009. The initial selection generated a high volume of articles, which were systematically reduced from over 20,000 to 197 articles. The exclusion criteria further included all publications with abstracts only, studies not touching on pre- or postoperative weight loss, or not documenting the postoperative outcome, duplicate studies, sample size less than 50. We also excluded case reports, case series, and studies using self-reported body weight data. This resulted in 16 articles. Additional supplementations of five articles from the reference list in the included articles brought it to a total of 21 publications. The assessment of methodological quality was done by using the critical appraisal skills program (CASP) tool.

**Figure 1:**
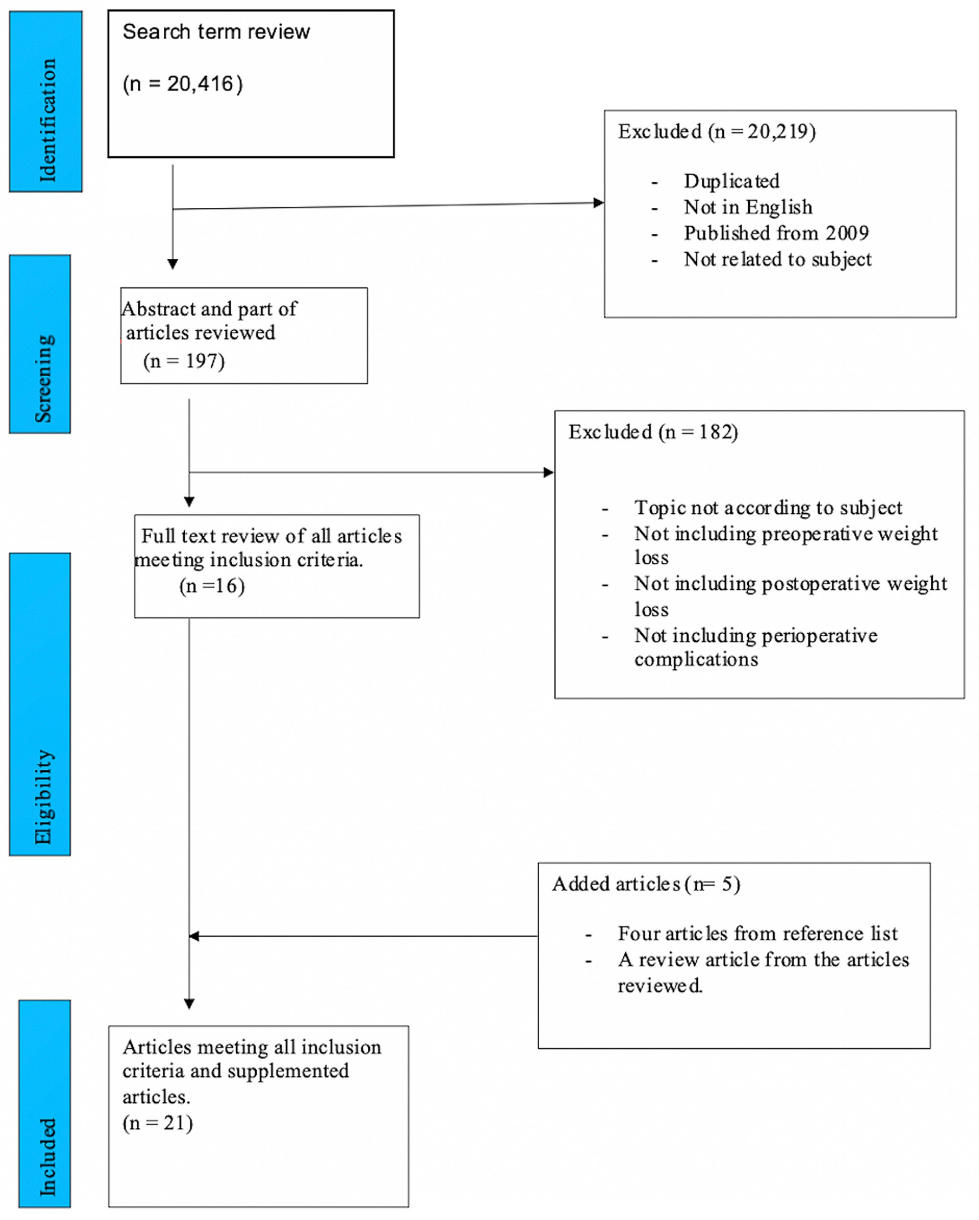
PRISMA consort diagram showing number of studies included and excluded in this review PRISMA - preferred reporting items for systematic reviews and meta-analyses

 Figure 1 presents a consort diagram depicting the method of exclusion and inclusion of publications [10].

Review

The observed results from bariatric surgery have generated enthusiasm among patients and physicians alike, resulting in the swelling of the surgical referral pool. This expanding pool of patients has led to the introduction of preoperative assessment guidelines to assist in patient selection for an optimized outcome from surgery [11, 12].

In the US, for instance, there are over 18 million adults that could qualify for bariatric surgery, but only about 1% of them end up undergoing surgery. Some of the contributing factors include social discrimination, lack of patient access to care, and funding challenges. A notable contributory factor is a requirement by some non-public-funded medical providers and bariatric centers to successfully complete preoperative weight loss programs. These programs are aimed at achieving weight loss in the range of 5-10%, hoping to optimize postoperative outcomes. Therefore, many bariatric centers came up with different eligibility criteria and management guidelines to balance needs and improve services [1, 7, 9].

The need to streamline management guidelines for patient selection in obesity surgery led the National Institute of Health (NIH) in 1991 to develop criteria and a standardized guideline for the treatment of severe obesity. While recommending self-motivated nonoperative weight loss efforts and robust multidisciplinary team evaluation, these criteria did not prescribe or mandate any specific type, degree, or duration of preoperative weight loss be completed. The 2005 updated report also did not require the completion of formal non-surgical obesity treatment as a condition precedent for eligibility to receive surgery [1].

Preoperative Weight Loss Effect on Postoperative Weight Loss

The practice of bariatric surgery has evolved from the period of identifying safe and effective surgery types to recent efforts at determining predictable factors for better postoperative outcomes. Historically, preoperative weight loss was advocated as contributory to the procedure's technical simplicity; however, it’s been advanced as potentially predictive of postoperative weight loss and patient compliance in recent decades. Many authors have sought to clarify this predictive role. The articles reviewed and their considered postoperative outcomes are shown in Table 1 [12-23].

**Table 1:** Summary of publications included in review RCT - randomized controlled trial; RNYGB - roux-en-Y gastric bypass; LRNYGB - laparoscopic roux-en-Y gastric bypass; SG - sleeve gastrectomy; LSG - laparoscopic sleeve gastrectomy; LAGB - laparoscopic adjustable gastric band; % EWL - percent excess weight loss; BMI - body mass index; LOS - length of hospital stay; PWL - postoperative weight loss; NA - not applicable

Author (reference)	Year	Study type	Procedure	Sample (n)	Variables assessed	Outcome / comments
Alami et al. [12]	2007	RCT	RNYGB	61	% EWL and operating room (OR) time	Improved % EWL, decreased operation time
Anderin et al. [9]	2015	Retrospective	RNYGB	22,327	Postoperative complication	Reduction of risk of complication
Blackledge et al. [3]	2016	Retrospective	LRYGB	300	Postoperative outcomes	No significant postoperative outcome
Becouarn et al. [14]	2010	Retrospective	RNYGB, SG,	507	% EWL	No difference in %EWL
Benotti et al. [11]	2009	Retrospective	RNYGB	881	Postoperative complication	Reduced complication
Conaty et al. [15]	2016	Retrospective	LRYGB, LAGB, LSG	717	EWL, comorbidity resolution, and readmission rates	No significant difference
Deb et al. [23]	2016	Retrospective	LRYGB, LAGB, LSG	200	Weight-loss attempts (WLA) and duration of weight loss on postoperative outcome	No significant difference in long term weight loss
Geber et al. [7]	2016	Retrospective	LRYGB, LAGB, LSG	200	Weight-loss attempts on EWL	No significant long-term outcome
Giordano et al. [16]	2013	Retrospective	LRYGB	548	Morbidity, mean excess weight loss	Reduced morbidity and improved weight loss
Hutcheon et al. [17]	2018	Retrospective	SG, LRYGB	355	EWL, operative time, and hospital length of stay (LOS)	Greater rate of postoperative EWL, reduced operative duration, and LOS
Jantz et al. [18]	2009	Retrospective	LRYGB	384	Weight loss (WL) and maximum WL on postoperative EWL	No correlation
Krimpuri et al. [19]	2017	Retrospective	RYGB, SG, LAGB	218	Postoperative EWL	Non- significant predictive value
Manning et al. [20]	2015	Retrospective	RYGB, SG	1456	Postoperative early weight loss on maximal weight loss	Maximal association at 3-6 months
Sherman et al. [21]	2015	Retrospective	LSG	141	% excess BMI change	No statistical difference
Solomon et al. [13]	2009	RCT	RNYGB	44	Postoperative EWL	Improved long term weight loss
Van Nieuwenhove et al. [22]	2012	RCT	LRYGB	298	Peri-operative complications, 30-day weight loss	Minor effect on variables
Livhits et al. [6]	2009	Systematic review	-	15 articles	Postoperative weight loss (PWL)	Positive effect
Cassie et al. [8]	2011	Systematic review	-	24 articles	PWL	No significant evidence
Ochner et al. [5]	2012	Systematic review	-	29 articles	PWL	No significant evidence
Geber et al. [2]	2015	Systematic review	-	25 articles	PWL	No significant evidence
Kim [1]	2017	Updated review	-	NA	PWL	No significant evidence

A study by Hutcheon et al. 2018 that implemented a four-week preoperative low-calorie diet to achieve preoperative weight loss investigated two cohorts of patients who underwent sleeve gastrectomy (SG; n=167) or roux-en-Y gastric bypass (RYGB; n=188). The first cohort achieved 8% excess weight loss (EWL; n=224), and the second cohort did not (n=131). Those who achieved 8% EWL had more EWL at postoperative months 3, 6 and 12 (42.3 ± 13.2% vs. 36.1 ± 10.9%, p<0.001; 56.0 18.1% vs 47.5 14.1%, p<0.001; 65.1 23.3% vs 55.7 22.2%, p<0.003, respectively). After conducting a regression analysis, the first cohort lost 7.5% more excess weight at postoperative month 12, supporting the advantageous position for preoperative weight loss [17].

Another study that focussed on the impact of preoperative weight amongst patients who underwent only laparoscopic roux-en-Y gastric bypass (LRYGB) categorized their patients into three weight loss groups. The first group A had patients (n=166) who lost less than 5% weight preoperatively; a larger second group B had patients (n=239), who lost between 5 to 10% and finally group C (n=143) patients who lost more than 10%. At one year follow up, those who lost most preoperative weight loss, group C, achieved significant postoperative weight loss (72.7%) compared to the least group A (63.1%; p=0.015) [16].

A further retrospective study encouraging weight loss before surgery but with no specific amount required evaluated postoperative weight loss in terms of BMI, % EWL, and percent total body weight loss (TBWL) in a review of 218 subjects. The mean age of study subjects was 44 years, 85% were female, and most of them (71%) had roux-en-Y gastric bypass with the remaining undergoing gastric sleeve (28%) and laparoscopic adjustable gastric band (1%). All patients had a mean 28% reduction in BMI (63.3% EWL and 29.1% TBWL) at one year post-operatively. As a single independent variable, pre-operative weight loss was a significant predictor of a one-year change in post-operative BMI (p=0.006). However, when other variables such as age, race, and gender were accounted for, this predictive value became non-significant (p=0.543) [19].

A few other studies have reported different outcomes from preoperative weight loss [3, 15]. An observational study to assess mandatory medically supervised preoperative weight loss (MPWL), which had a compulsory requirement of at least 10% excess body weight loss before surgery, compared postoperative weight loss outcomes. Patients were divided into two cohorts based on participation in the MPWL program or non-participation. Inclusion criteria was met by 717 patients, out of which 465 underwent surgery without a preoperative weight loss requirement, and 252 participated in the MPWL program. One year after surgery, there was no marked difference in average EWL percentage between non-participants (58.6%) and MPWL participants (59.1%; p=0.84) [15].

Deb et al. 2016 retrospectively reviewed 200 morbidly obese patients who underwent LRYGB, laparoscopic adjustable gastric band (LAGB), or laparoscopic sleeve gastrectomy (LSG). Among them, 154 completed the survey, requiring prior weight loss attempts (measured during preoperative clinic assessment), and the impact of these attempts was evaluated against postoperative weight loss outcomes. BMI and percentage of excess weight loss were used to evaluate weight loss. The mean number of weight loss attempts (WLAs) before surgery was 3.5 ± 0.2 attempts, achieved over a 15 year period (average duration of 15.2 ± 1.1 years). Preoperative BMI and weight loss duration had a negative relationship (r=-0.2637, p=0.0025), and overall there was no significant difference for preoperative BMI or mean 12-month EWL percentage among any WLA groups. The evidence suggests that long-term weight loss outcomes are not impacted significantly by the number and duration of preoperative dietary attempts. Therefore, recommending additional preoperative WLA may not be an effective strategy at improving patients’ chances of weight loss [23].

Another retrospective review in a single institution reported no significant changes between preoperative excess weight change percentage and weight loss outcomes at 24 months. In their study, patients were stratified into quartiles based on the percentage of excess weight gain (0-4.99% and ≥ 5% EWG) and the percentage of excess weight loss (0-4.99% and ≥5 % EWL). They all underwent LRYGB [3].

Interestingly, the only included study involving those that specifically had only laparoscopic sleeve gastrectomy was a retrospective analysis of 141 patients based on preoperative weight loss or gain. After one year, the analysis revealed that 72 of them lost weight and 64 gained weight preoperatively. Only about six of them maintained the weight. No demonstrable difference was found between both groups in postoperative weight loss or operative time at one year [21].

In a unique study involving 537 patients who had a primary bariatric procedure by the same surgeon with similar preoperative multidisciplinary care over a period of 12 months and follow-up 48 months after surgery, no correlation was evidenced between pre- and postoperative weight loss regardless of the surgical technique performed. Gender or initially recorded BMI class (whether greater or lower than 50kg/m^2^) did not impact the excess weight loss. Though patients varied in their measured weight changes before surgery, no trend of relationship to the outcome could be established. However, this was a retrospective study that reviewed disproportionately more gastric bypass (n=381) procedures compared to the sleeve gastrectomy (n=29) [14].

As at the time of this review, four randomized clinical trials focussed on the significance of preoperative weight loss. Two of them involved laparoscopic gastric band procedures and were not included in this review. In one of the very few RCTs, Alami et al. 2007 reviewed 100 patients undergoing laparoscopic gastric bypass categorized preoperatively to either a weight loss group with a 10% weight loss requirement and a non-weight loss group. The weight loss (WL) group's (n=26) average initial BMI was 48.7kg/m^2,^ while the non-weight loss (NWL) group's BMI was 49.3kg/m^2^. After three months, results demonstrated significant excess weight loss in the weight loss group compared to the non-weight loss group (44.1% vs. 33.1%, p<0.026). However, this significant improvement was not replicated at six months postoperative period as recorded excess weight loss stood at 53.9% and 50.9% (p was not significant). The study highlighted the calculated time from initial consultation to surgery was 5.4 months and 5.2 months; however, it did not go further to investigate whether there is any implication to this interval to surgery. They concluded that there is a derivable short-term advantage regarding improved postoperative weight loss if patients lose weight preoperatively. Beyond six months, this advantage appears unsustainable. Perhaps the limitation to this finding might be related to the available small sample size number at six months, which stood at 37 [12].

In 2009, Solomon et al. conducted a reanalysis of the same RCT above over a 12 month period. At this time, there were 44 patients in the two groups with very significant contrasting weight changes. The NWL group rather gained 1.1% (p<0.007) while the WL group, on average, lost 8.2% of their excess body weight preoperatively. Despite this, there was no demonstrable significant statistical difference in both arms of the study in terms of weight, BMI, and excess weight loss. But when they were arbitrarily categorized into two groups, using minimum preoperative excess body weight loss of 5% as the yardstick, those who met the target (n=19) had statistically significant lower BMI and a higher percentage of excess body weight loss at one-year post-surgery. The study surmised that preoperative weight loss should be encouraged as it would improve long term weight loss. The relatively low number of patients was a limitation to this study [13].

An additional RCT by Van Nieuwenhove et al. 2012 reported no difference in postoperative weight loss between RYGB patients. They compared patients who achieved weight loss preoperatively on an 800 kcal per day all-liquid meal diet vs. control (4.9±3.6 kg) over a 30 day period [22]. The short follow-up, however, was the limitation of the study.

A systematic review published in 2009 comprising 909 screened reports spanning between 1988 and 2009 revealed that a mean difference of 5% excess weight loss in one-year postoperative could be achieved from preoperative weight loss. They arrived at this conclusion after excluding low-quality studies from their review. The review noted that a further advantage of losing weight preoperatively would be in determining compliance amongst patients. Howbeit, the drawbacks with the review stem from the heterogeneous composition of the studies and methods of defining weight loss. Some of the weight-loss parameters include the percentage of EWL or percentage of the total weight or a change in pounds or BMI. A similar difficulty was witnessed in determining the timing of preoperative weight value. These could create ambiguity in concluding a possible positive effect of preoperative weight loss [6].

Three systematic reviews published after 2010 did not show sufficient evidence that preoperative weight loss confers improved postoperative weight loss outcome [5, 7, 8]. In the first review article by Cassie et al. 2011, 24 studies reporting postoperative weight loss were analyzed. Nine out of them involving 2,177 patients demonstrated a significant improvement in postoperative weight loss, while the remaining 15 reports involving a much larger patient population (3,252 total patients) noted a contrary outcome. The majority of the studies measured weight loss by the percentage of EWL. Analyzing weight loss at 12 months, the preoperative weight loss patients witnessed a 62-78.4% reduction in EWL and the non-preoperative weight loss patient 60-76% EWL. Combining data from their studies over one and two years, the preoperative weight loss patients group reported less EWL percentage (69.0% ± 7.1%, 66.7% ± 2.7%) compared to the non-preoperative (70.7% ± 5.7%, p=0.67; 72.0% ± 6.3%, p<0.001), respectively. Essentially, only three studies were vital in arriving at the above finding. The review concluded little evidence is available to validate the practice of ensuring preoperative weight reduction before bariatric surgery. [8]

The other two subsequent reviews by Ochner et al. 2012 and Geber et al. 2015 arrived at a similar conclusion to Cassie et al. [5, 7]. The review by Geber et al. 2015, interestingly included data from three previously published systematic reviews and the largest cohort study from the Scandinavian Obese Subjects Registry (SOSReg). Of note, the SOSReg study analyzed data on 22,327 patients majority of whom underwent LRYGB (96%). The limitation here was the inability to provide data on long term weight loss outcomes [7].

The most updated review published by Kim in 2017 analyzed four RCTs, three systematic reviews, and several case series concluded that most recent studies had not proven any clear benefit of weight loss before surgery [1]. Particularly, many of the recruited uncontrolled case studies demonstrating the advantage of preoperative weight loss were published more than 10 years ago. A deducible reality is that achieving set targets of either 10% or even 5% preoperative weight loss for the severely obese population may not be an easy and timely objective irrespective of the motivation either for bariatric surgery or general health efforts. The review further observed the latest position statement from the American Society for Metabolic & Bariatric Surgery (ASMBS) regarding the impact of insurance mandated preoperative weight loss, demonstrating no clear evidence on postoperative weight loss outcomes [24]. The position statement further argued that just like every other elective surgical procedure, there should be no attached precondition for weight loss or proof of lifestyle [1, 24].

Operative Outcomes and Postoperative Complications

Some of the other documented outcomes impacted by preoperative weight loss include operative time, length of stay, postoperative complications, readmission rates, and resolution of co-morbidities. Several studies have reported varied preoperative weight loss effects on intraoperative and postoperative outcomes [5, 9, 11-13]. Alami et al. 2007 had noted that pre-surgery weight loss led to a reduction in liver size, helping to enhance visibility during surgery and invariably reducing the technical complexity of the bariatric procedure. Ultimately, this led to reduced operation time and complications [12].

The largest Scandinavian Obesity Registry (SOREG) cohort study of over 22,000 people who underwent gastric bypass had a median preoperative weight loss change of -4.8% with corresponding values of 0.5, -4.7, and -9.5% when classed in 25th, 50th, and 75th percentile respectively [9]. The complication rate was noted in 9.1% of them, but this was reduced by 13% amongst the 75th percentile group compared to the 25th percentile group of preoperative weight loss. Correspondingly, less pronounced risk reductions favored the 50th percentile group than the 25th percentile. There was a statistically significant risk reduction attributable to preoperative weight loss for all analyzed complications [9].

It has been documented that more of the postoperative complications appear to occur with open procedures compared to the laparoscopic types. A retrospective study of 881 patients who underwent gastric bypass, with 466 of them undergoing open and 415 laparoscopic, reported respectively total and major complications (p<0.01 and p=0.03). Notably, the majority of them were older male patients with higher BMI (p<0.001). On regression modeling, increased preoperative weight loss could predict reduced complications [11].

Further studies have also noted peri-operative advantages derivable from preoperative weight loss [13, 17]. In the earlier mentioned study requiring four-week LCD amongst patients who had LSG and RYGB divided into two cohorts of achieving 8% EWL or not. Those who lost 8% EWL had reduced operative time and shorter hospital stay (117 minutes vs. 125 minutes, p<0.061; 1.8 days vs. 2.1 days, p<0.006). However, the readmission or re-operation rates were not significantly different [17].

The study by Giordano et al. involved RYGB with a division of patients into three groups based on preoperative weight loss percentage of <5% (group 1, n=166), >5% to 10% (group 2, n=239), and >10% (group 3, n=143), showed a significant comparative reduction in operative time (mean ±SD; 104.43±36.40 min in group 1, 80.08±23.07 min in group 2, and 76.99±23.23 min in group 3; p<0.001). A similar comparative significant reduction in length of stay in the hospital was observed (3.33± 3.22 days in group 1, 2.10±2.77 in group 2, and 1.87±1.44 in group 3; p<0.001). The same trend was observed in the overall postoperative morbidity rate [16].

Blackledge et al. 2016 had a different observation in their retrospective study of 300 patients with LRYGB with no hugely divergent demographic differences. The study stratified the patients into quartiles of excess weight gain percentage (0-4.99% and ≥5% EWG) and percentage of excess weight loss (0-4.99% and ≥5% EWL). They showed that despite the higher rate of complications in the weight gain group (12.5 vs. 4.8, p=0.29), this was not statistically significant in the overall peri-operative and postoperative outcomes. Remarkably, there was a delayed time to operation for patients who gained or lost 5% excess body weight (p<0.001). Therefore the study suggests the time to operation may be increased by insisting on unvalidated preoperative weight loss requirement [3].

Additional studies by Conaty et al. 2016 and Sherman et al. 2015 did not find significant postoperative outcomes as well [15, 21]. The observational study by Conaty et al. was conducted to assess the efficacy of mandatory medically supervised preoperative weight loss (MPWL); out of 717 patients, 465 underwent surgery without following the requirement while 252 participated. The readmission rates at 30 days (3.4 vs. 6.4%, p=0.11) and 90 days (9.9 vs. 7.5%, p=0.29) postoperatively were not statistically different from the non-participants and MPWL patients, respectively. A year post-surgery, a similar proportion of patients (67.1% non -participants and 62,5% of MPWL participants) showed a resolution with regards to at least one of the five identified co-morbidities (p=0.45) [15]. Sherman et al., in their retrospective review of patients who underwent LSG alone, did not demonstrate any significant statistical difference in the percentage postoperative excess BMI and length of stay studied [21].

The systematic review by Cassie et al. 2011 assessed hospital length of stay (LOS) from seven studies. Only five of these reported numerical values, and they all analyzed gastric bypass patients. The LOS ranged from 2.2 to 4.3 days for the preoperative weight loss group and 2.3 to 6.0 days for the non-preoperative weight loss group. The combined mean LOS was not significantly different among the preoperative weight loss (3.34 ± .83 days) and the non-preoperative weight loss (3.98 ±1.49 days, p=0.05) patients [8]. Livhtis et al. 2009, in their review, could only identify two high-quality studies that still didn't find significant differences between the two groups [6].

On assessing operative time, Cassie et al. 2011 relied on the data from the RCT by Alami et al. 2007. This showed a significantly shorter total operating room time in the preoperative weight loss group, 220.2 minutes vs. 257.6 minutes for the non-preoperative weight loss group. The six out of the eight trials that focussed on LRYGB, albeit retrospective, showed improved operative times for the non-weight loss group (119.7 to 212.5 minutes) vs. 104.9 to 176.3 minutes in the weight loss category. The aggregate results showed preoperative weight loss resulted in 12.5 minutes of shorter procedures. The review had significant heterogeneity among the studies and particularly observed that most studies failed to specify how operative time was measured [8]. A meta-analysis of the operative times was conducted by Livhtis et al. 2009 using only three high-quality studies. The study had significant heterogeneity (p=0.07), but on the whole, preoperative weight loss was found to reduce operative time by 23 minutes (95% CI: 13.8-2.8) [6].

In summary, available data, including recently published systematic reviews and a most up-to-date evidence base review, show no conclusive evidence that pre-specified weight loss before surgery improved postoperative outcomes [1, 8, 12].

## Conclusions

Many bariatric centers practice the requirement of ensuring pre-specified weight loss before receiving surgery; however, many of the recent high-quality reviews are not conclusive of the evidence supporting this practice. Therefore, this literature review identifies the challenge of continuing such recommendation and calls for a re-evaluation of our selection process. While modest weight loss is potentially advantageous regardless of intention, setting a target before bariatric surgery should not lead to delay or denial of beneficial treatment. It would seem reasonable to allow the multidisciplinary team of bariatric providers to drive the practice protocols to serve their patients' best interests. The need to clarify the controversy surrounding preoperative weight loss requirements in a large-scale, multi-center, highly powered study cannot be overstated. More focus should be placed on other procedures beyond roux-en-Y gastric bypass that are increasingly gaining momentum.

An identified area that needs to be explored is perhaps the contribution of the duration of severe obesity to postoperative weight loss outcomes. How long a patient may have lived with a severe form of obesity before receiving bariatric surgery may need to be appropriately identified, categorized and its effect analyzed against postoperative weight loss outcomes. This duration of obesity is entirely different from a patient's age as the development of the severe form of obesity can occur at different times of a patient’s life.
